# The application of post-translational modification oriented serum proteomics to assess experimental diabetes with complications

**DOI:** 10.1371/journal.pone.0206509

**Published:** 2018-11-05

**Authors:** Han-Min Chen, Lin-Chien Lee, Kuang-Yu Hu, Wei-Jern Tsai, Cheng Huang, Hui-Jen Tsay, Hui-Kang Liu

**Affiliations:** 1 Department of Life Science, Fu Jen Catholic University, New Taipei city, Taiwan, ROC; 2 Department of Physical Medicine and Rehabilitation, Cheng Hsin General Hospital, Taipei, Taiwan, ROC; 3 Department of Biochemistry, National Defense Medical Center, Taipei, Taiwan, ROC; 4 Division of Chinese Medicine Literature and Informatics, National Research Institute of Chinese Medicine, Ministry of Health and Welfare, Taipei, Taiwan, ROC; 5 Department of Biotechnology and Laboratory Science in Medicine, National Yang-Ming University, Taipei, Taiwan, ROC; 6 Department of Earth and Life Sciences, University of Taipei, Taipei, Taiwan, ROC; 7 Institute of Neuroscience, Brain Research Center, school of life science, National Yang-Ming University, Taipei, Taiwan, ROC; 8 Division of Basic Chinese Medicine, National Research Institute of Chinese Medicine, Ministry of Health and Welfare, Taipei, Taiwan, ROC; 9 Ph.D. Program in Clinical Drug Development of Chinese Herbal Medicine, Taipei Medical University, Taipei, Taiwan, ROC; Stellenbosch University, SOUTH AFRICA

## Abstract

Proteome analysis of serum from type 2 diabetics with complications may lead to the discovery of diagnostic or prognostic biomarkers. To circumvent the principal barrier of serum proteomics, our investigation aimed to evaluate whether a study of post-translational modification enriched serum proteins could be valuable for the discovery of biomarkers or metabolic pathways related to type 2 diabetes pathogenesis. Type 2 diabetes was induced from high-fat diet fed Sprague Dawley rats with streptozotocin injection. Once diabetic status was confirmed, serum samples from either fasted healthy or diabetic rats were pooled and profiled by two-dimensional difference gel electrophoresis or comparative 2D electrophoresis after protein enrichments using immobilized metal ion, concanavalin A, and lentil affinity chromatography, respectively. Differential expressed proteins were identified and the associated networks were established by an Ingenuity Pathway Analysis. As a result, induced rats became severe diabetic and accompanied by hyperlipidemia, fatty liver, and glomerular hypertrophy. There were 3 total, 14 phosphorylated and 23 glycosylated protein targets differentially expressed. Proteins could be linked to HNF4A, HNF1A, and NF_κ_B transcriptional factors and antigen presentation, humoral immune response, and inflammatory response pathways. Predicted organ toxicity in kidney, heart, and liver matched with our histopathological results. In conclusion, post-translational modification based serum protein enrichment could be a valuable approach to enhance the resolution of serum proteomics without depleting potentially valuable abundant proteins. Our results also indicated the potential association of the hepatic secretome and hepatocyte nuclear factors in the pathogenesis of type 2 diabetes and its complications.

## Introduction

Diabetes is a chronic disease caused by hyperglycemia due to absolute insulin deficiency or abnormal insulin function [1]. In contrast to the non-obesity-related type 1 diabetic population, type 2 diabetic (T2D) patients account for approximately 95% of the diabetic population. The incidence of the disease is increasing rapidly and is expected to affect 300 million people by the year 2025 [2]. Although diabetes itself is not lethal, chronic hyperglycemia in diabetes could lead to deleterious effects in multiple organs and cause diabetic complications.

Glycosylated hemoglobin (HbA1c%) is positively correlated to serum advanced glycated end products (AGEs), which are formed by the Maillard Reaction [3, 4]. Through the interaction of AGEs and receptors of AGEs (RAGEs), oxidative stress and inflammatory responses are expected to be induced and to lead to different extents of tissue damage. As a result, the prognosis of the risk of developing diabetic complications is based on the level of (HbA1c%), which is also one of the criteria for diabetes, according to the American Diabetes Association 2010 Standards of Medical Care in Diabetes [5].

In addition to conventional HbA1c% monitoring, the application of serum proteome profiling potentially could facilitate T2D diagnosis, prognosis, and therapeutic efficacy evaluation [6–8]. Furthermore, serum proteomics may lead to the elucidation of the T2D complication pathogenesis [9]. However, the wide variety of serum protein concentrations limited the unbiased identification of differential expressed protein targets. Therefore, low abundance proteins are usually under detection [10]. Depletion of abundance proteins may increase the sensitivity of certain proteins but overlooks the contributions of high abundance proteins in pathogenesis of T2D [11].

Post-translational modification (PTM) is usually defined as the covalent and enzymatic modification of proteins after protein translation. Altered PTM among serum proteins could affect normal protein functions and related to the pathogenesis of a disease. For example, protein glycation levels in diabetics are increased under hyperglycemic condition. As a result, the bioactivity of important metabolism regulating hormones, like insulin, could be further affected by protein glycation in diabetics [12]. In addition to glycation, altered glycosylation and phosphorylation of serum proteins in diabetes by enzymes that regulate PTM have also been reported [13–15]. Therefore, a comprehensive serum proteomics from the sub-fractions of protein enriched with specific post-translational modifications might provide valuable lessons for understanding pathophysiology of disease.

In the present investigation, we aimed to profile serum samples with or without PTM protein enrichment. Besides, we would like to network our identified protein targets to explore potential biomarkers or identify disturbed metabolic pathways which are related to the pathogenesis of diabetes and its complications. The experimental T2D model is induced by a single injection of low dose streptozotocin under high-fat diet (HFSTZ) in rats. This model provides the typical pathological features of T2D including peripheral insulin resistance, impaired insulin secretion, and glucose intolerance [16, 17]. Additionally, the responsiveness towards various hypoglycemic drugs in this model is well-preserved.

## Materials and methods

### The preparation of type 2 diabetic (T2D) rats induced by a high fat diet plus a single streptozotocin injection (HFSTZ)

Sprague-Dawley (SD) rats were purchased from the National Yang-Ming University Animal Center, Taipei, Taiwan. All experimental procedures followed the Guide for the Care and Use of Laboratory Animals (NIH publication, the 8^th^ edition, revised in 2011) and were approved by the Animal Research Committee at the National Research Institute of Chinese Medicine. Adult male SD rats weighing 250–350 g were housed under controlled conditions (i.e., a 23°C room temperature, controlled humidity, and a light/dark cycle of 12/l2 h). The preparation of the T2D rats was based on the method reported by Sirnivasan et al. with some modifications [16, 17]. Briefly, beginning at the age of 8 weeks, rats were fed a high-fat chow (high-fat diet) made from standard chow supplemented with extra lard (20%, w/w), cholesterol (1%, w/w), and cholic acid (0.1%, w/w). After 2 weeks of dietary manipulation, the rats on the high-fat-diet received a single intraperitoneal (i.p.) injection (50 mg/kg body weight) of streptozotocin (Sigma, St. Louis, USA). Four weeks post-injection, the rats with a blood sugar greater than 300 mg/dl (16.7 mM) were regarded as the HFSTZ-induced T2D rats.

### Intravenous Glucose Tolerance Test (IVGTT)

To evaluate changes in glucose tolerance between the healthy control (n = 8) and the T2D rats (n = 8), the rats were fasted overnight and a glucose solution was injected (1 g glucose/kg body weight) intravenously via the tail vein. Blood sugar levels were measured immediately (0 min), and at 15, 30, 60, and 120 min throughout the test.

### Serum biochemical analysis

After overnight fasting, blood samples from the healthy control (n = 8) and the T2D rats (n = 8) were collected from the tail vein of rats anesthetized with pentobarbital (30 mg/kg of body weight, i.p.). Blood glucose was immediately measured using a commercial glucometer (Bioptik Technology, Taipei, ROC). Prepared serum samples were then subjected to analysis using a FUJI DRI-CHEM clinical chemistry analyzer to determine serum triglyceride, total cholesterol, and glutamate pyruvate transaminase (GPT) concentrations. In addition, serum insulin was measured by using an ELISA kit (Millipore, Bedford, USA). Both the homeostasis model assessment (HOMA) for insulin resistance [HOMA-IR = Fasting blood glucose (mM) x Fasting insulin (μU/mL) / 22.5] and HOMA for beta-cell function [HOMA-B = 20 × Fasting insulin (μU/mL) / Fasting blood glucose (mM) - 3.5] were calculated.

### Pathological examinations

At the end of the experiment, pentobarbital (90 mg/kg of body weight, i.p.) overdose rats were sacrificed in order to harvest livers and kidneys. A portion of each organ was fixed in 4% (w/v) paraformaldehyde for paraffin sectioning. For pathohistological analysis, hematoxylin and eosin (H&E) staining was used to illustrate the overall morphology. Periodic Acid-Schiff (PAS) staining was performed to determine the glycogen content. Masson’s trichrome stain was used to determine cellular glycogen (a purple color). To determine the relative size of the glomerulus in the kidney sections stained by PAS, Image J (the National Institutes of Health, USA) was employed to calculate the total pixels in the region of each glomerulus from control and T2D rats.

### Preparation of protein samples

Sera from the healthy control (n = 8) and T2D (n = 8) rats were processed as follows: an equal volume of all sera in each group was mixed to generate a serum pool without any pre-process. To analyze the total proteome, serum pools were subject to 2-DE analysis using a difference gel electrophoresis (DIGE) kit (GE healthcare, Waukesha, WI, USA). Healthy control serum pool labeled with Cy3 dye, T2D serum pool labeled with Cy5 dye, and Healthy plus T2D equal serum pool labeled with Cy2 dye as internal standard. To analyze PTMs, serum pools were subjected to phosphoprotein and glycoprotein purification. Phosphoproteins were purified using an immobilized metal affinity chromatography (IMAC) resin (PhosPRO phosphoprotein purification kit, PP05-2C, Visual Protein, Taipei, Taiwan, ROC). Approximately 100 μg of phosphoproteins were recovered from each 80 mg fraction of the serum pool. Glycoproteins were purified using either the Concanavalin A (ConA) resin (GlycoPRO glycoprotein enrichment kit, GP05, Visual Protein) or the Lentil resin (L4018, Sigma-Aldrich, St. Luis, USA). Approximately 50 μg of glycoproteins were enriched from each 1 mg of fraction of the serum pool. Purified phosphoproteins and glycoproteins were harvested using Trichloroacetic acid (TCA) precipitation and quantitated using a Bicinchoninic acid (BCA) protein quantitation method, as previously described [18].

### 2-DE experiments

2-DE was performed as previously described with minor modifications [4, 18]. Briefly, TCA-precipitated proteins were dissolved in standard 2-DE rehydration buffer containing 8 M urea, 2% CHAPS, 0.5% IPG buffer, and 18 mM DTT. Protein samples were loaded on IPG strips (13 cm, pH 3–10 Nonlinear, GE Healthcare) using a rehydration loading method. After 12 h of rehydration on the Ettan IPGphor II system (GE Healthcare), Isoelectric Focusing (IEF) was performed for the required number of voltage-hours, which was determined using the IEF optimizer (Visual Protein) and calculator (www.visualprotein.com). The IEF voltage program for 2D-DIGE: current per strip was 100mA. 500 V for 1hr, 1000 V for 1hr, 8000 V to a total of 37500vh for total 15.14 hr. The IEF voltage program for 2DE-IMAC: current per strip was 100mA. 500 V for 1hr, 1000 V for 1hr, 8000 V to a total of 6400vh for total 14.8 hr. The IEF voltage program for 2D-Con A/2D-Lentil: current per strip was 100mA. 500 V for 1hr, 1000 V for 1hr, 8000 V to a total of 13312 vh for total 15.14 hr. The IPG strips were run in the second dimension 12.5% SDS-PAGE gels using the SE-600 electrophoresis system (GE Healthcare). The 2-D gels labeled with Cy dyes or stained with the SYPRO Ruby protein stain (Invitrogen, Waltham, MA, USA) were imaged using the Typhoon Trio plus laser gel scanner (GE Healthcare). The image volume of the protein targets on the 2-D gel images was analyzed using the ImageMaster 2D Platinum software (GE Healthcare). The relative abundance of each protein was then calculated by normalizing the volume of a protein target to the sum of volume of all protein spots on the same 2-D gel image.

### Mass Spectrometry (MS) protein identification

MS sample preparation was performed as reported previously [19]. Briefly, after re-staining the SYPRO Ruby stained gels using VisPRO 5 Minutes Protein Stain Kit (Visual Protein), protein targets on the 2-D gels were manually excised as approximately 1 mm diameter gel spots and processed according to a standard MS sample preparation protocol. In-gel digestion of the excised gel spots was performed using MS-grade Trypsin Gold (Promega, Madison, WI, USA) overnight at 37°C. Tryptic digests were extracted using 10 μL Milli-Q water, followed by three extractions totaling 20 μL 0.1% trifluoroacetic acid (TFA). The pooled extracts were dehydrated in a vacuum concentrator and then dissolved in 1 μL of buffer containing 5% acetonitrile and 0.5% TFA. The prepared MS samples were analyzed using an electrospray quadrupole time-of-flight (ESI-QUAD-TOF) MS analyzer (Q-TOF 2, Waters). All peak lists were generated from individual spectrum using MassLynx (version 4.0 SP4) with a signal to noise (*S/N*) threshold of 2. Peak lists were then submitted to the Mascot search engine for MS/MS Ions Search function (www.matrixscience.com). The search parameters were as follows: NCBI nr (version: 20090619~20100529, 9111587~11111565 sequences; 3119984970~3786451707 residues) database, *Rattus* (226105 sequences) taxonomy; trypsin enzyme, carbamidomethylation (cysteine) fixed modification, deamidation (asparagine and glutamine) and oxidation (methionine) variable modification, precursor ion mass tolerance of ± 0.2 Da, MS/MS tolerance of ± 0.2 Da, and one missed cleavage. The peak lists from mass spectra of individual MS protein identifications were shown in the **Web Enhanced Object**. False discovery rate (FDR) was undetermined during MS protein identification.

### Systems biology analysis

The systems biology analysis was performed using the Ingenuity Pathway Analysis (IPA, Ingenuity Systems, www.ingenuity.com). In order to investigate the possible biological events involved, the identities (GI number) of differentially expressed proteins, phosphoproteins, and glycoproteins and the corresponding protein expression ratio were uploaded into IPA. Each gene identifier was mapped to its corresponding gene object in the Ingenuity Pathways Knowledge Base (IPKB). These genes, called focus genes, were overlaid onto a global molecular network developed from information contained in the IPKB. Networks of these focus genes were then algorithmically generated based on their connectivity. The functional analysis of a network identified the biological functions and/or diseases that were most significant to the genes in the network. A Fisher’s exact test was used to calculate a P-value to determine the probability that each biological function and/or disease assigned to a network was due to chance alone.

### Statistics

The significance of various treatments was determined using a Student’s unpaired t-test. The results were expressed as the mean±standard error of the mean (SEM). Differences were considered significant when *p<0.05, **p<0.01, and ***p<0.001.

## Results

### Biochemical and pathological characteristics of HFSTZ-induced T2D rats

The comparison of biochemical parameters between the control and T2D groups is summarized in Table 1. After the onset of T2D, there was a 20% reduction of body weight in the T2D group (*p*<0.05). However, the fasting blood sugar levels in the T2D rats increased 2.5-fold (*p*<0.001) when there was no difference in fasting insulin concentration. As a result, HOMA-IR increased 4-fold (*p*<0.001) and HOMA-B decreased 70% (*p*<0.001) in the T2D rats. In terms of serum lipids, the levels of both serum triglyceride and total cholesterol increased 1.5-fold (*p*<0.05) and 3-fold (*p*<0.05) in the T2D rats, respectively. Finally, the serum GPT concentration and HbA1c% increased 3-fold (*p*<0.01) and 2-fold (*p*<0.001) in the T2D rats, respectively.

**Table 1:** Biochemical characteristics of control and diabetic group.

	Control (standard diet)	Type 2 diabetes (High fat diet)
**Body weight (g)**	369 ± 16.8	295 ± 11.4*
**Glucose (mg/dl)**	134.3 ± 2.3	338.8 ± 17.7 ***
**Insulin (ng/ml)**	0.28 ± 0.12	0.38 ± 0.11
**HOMA-IR**	2.8 ± 0.07	12 ± 0.63***
**HOMA-B**	13.4 ± 0.07	3.8 ± 0.35***
**Triglyceride (mg/dl)**	81.6 ± 6	123.4 ± 21.8*
**Total cholesterol (mg/dl)**	60.5 ± 3.3	202.6 ± 78.2*
**GPT (mg/dl)**	11 ± 1.6	28 ± 2.5**
**HbA1c (%)**	3.14 ± 0.11	7.8 ± 0.32***

Data represented as mean±SEM (n = 8).

*p<0.05

**p<0.1, and

***p<0.001 comparing with that of control group.

**Fig 1:**
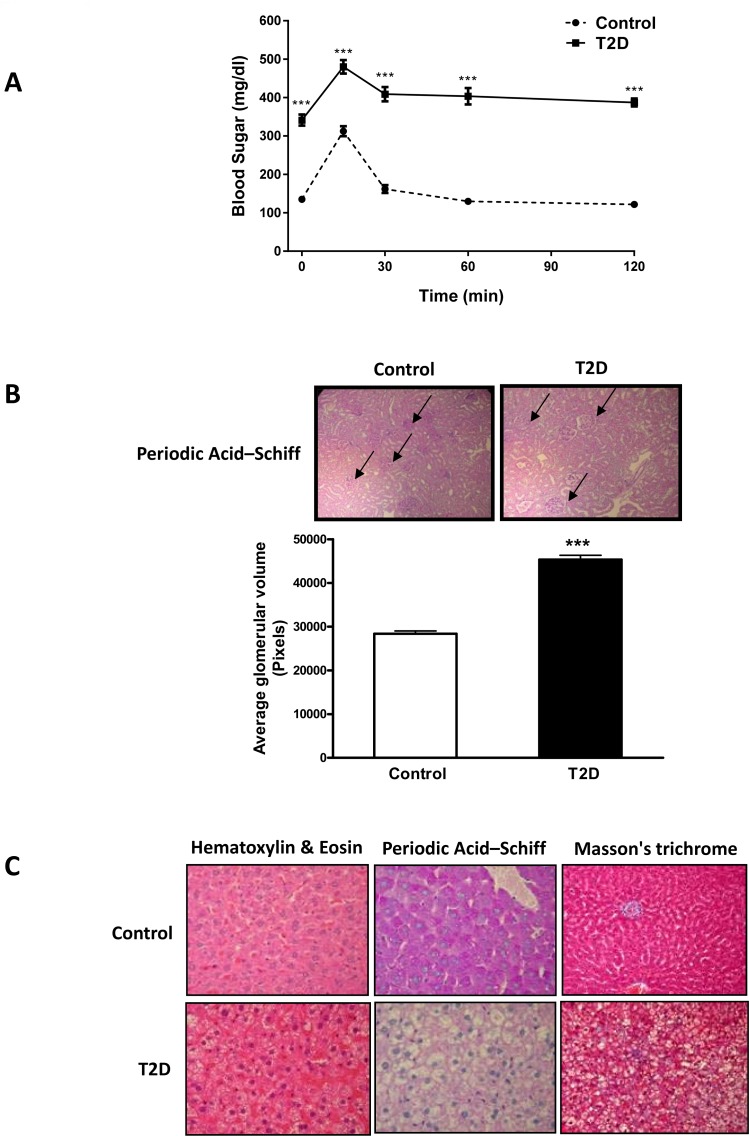
Pathological characteristics of HFSTZ-induced T2D rats. (A) Evaluation of the extent of glucose intolerance in the T2D rats induced by HFSTZ. Data are the mean±standard error of the mean (SEM; n = 8). *** = *p*<0.001 versus control group. (B) Evaluation of the glomerulus volume between the control rats and T2D rats. Representative images of the glomerulus stained with Periodic Acid–Schiff (PAS) are illustrated. Glomerular volume was quantified by the pixels of a randomly selected glomerulus. Data are the mean±SEM (n = 300 from three rats). *** = *p*<0.001 versus control group. (C) Evaluation of the extent of hepatic steatosis in the T2D rats. Liver sections of control or T2D rats were stained with hematoxylin & eosin, PAS, or Masson's trichrome. Representative images are illustrated correspondingly.

In terms of pathological features, glucose tolerance was significantly impaired in the T2D rats, as shown in Fig 1A. In addition, a computer-based glomerular volume estimation indicated that the relative glomerular volume in the T2D rats increased about 1.5-fold (*p*<0.01) compared to the volume in the control rats (Fig 1B). Finally, in Fig 1C, the morphology of diffuse hepatic microvesicular steatosis in the liver sections from the T2D rats was evaluated by a loss of cytoplasmic eosin stain. On the other hand, the hepatic glycogen content appeared to decrease due to the lack of PAS staining signal. Moreover, the presence of liver fibrogenesis was assessed in the T2D rats by the accumulation of cytoplasmic collagen staining with Masson’s trichrome stain.

### Total proteome analysis

**Fig 2:**
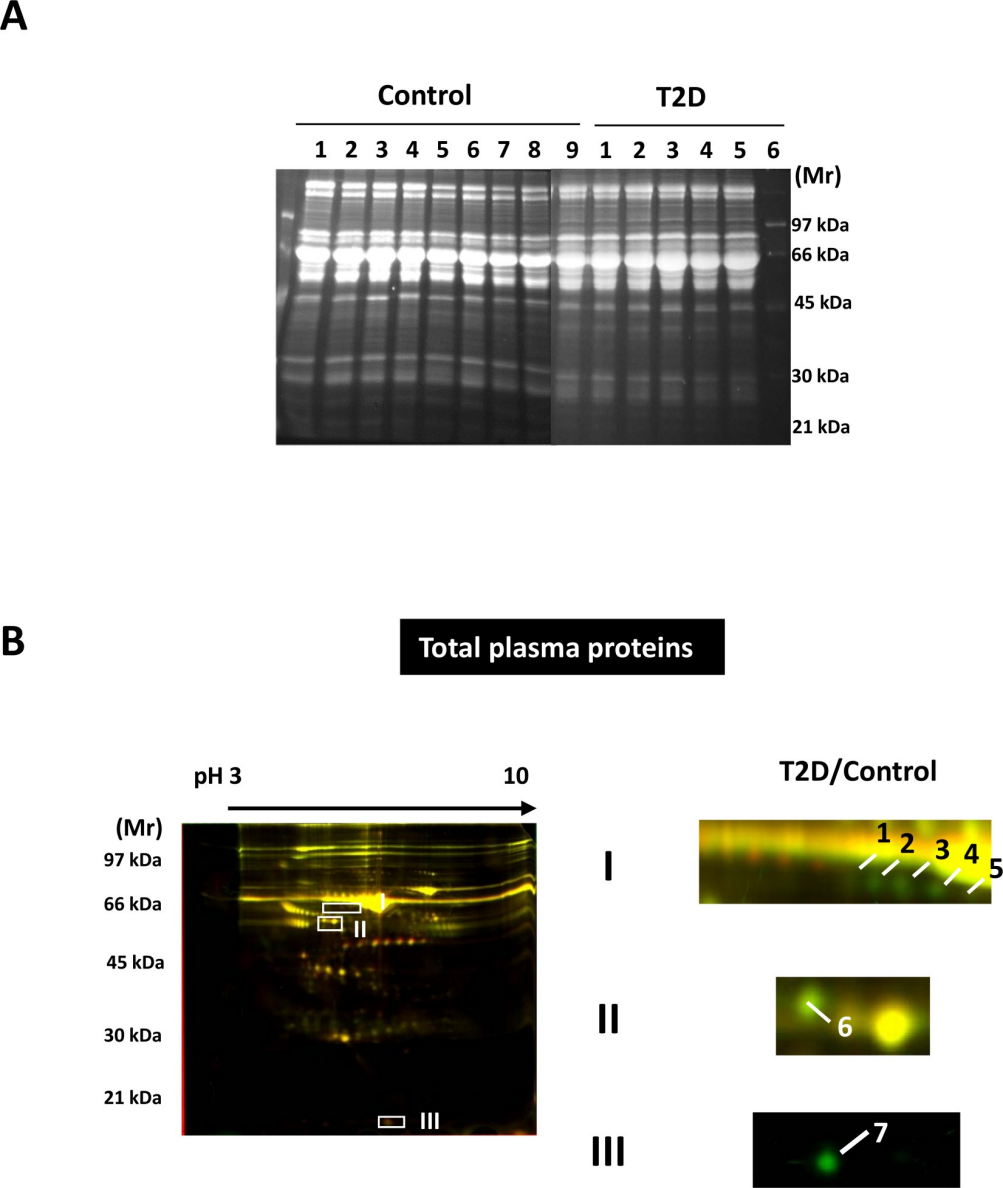
Differentially expressed proteins expressed in the serum proteomes of the control and T2D rats. (A) 1-DE analysis of the sera before pooling. Proteins (30 mg) were subject to analysis using 12.5% SDS-PAGE gels. Gels were stained with SYPRO Ruby as described in the experimental procedures. (B) The overlaid 2-D DIGE images for the control and T2D serum pools. Proteins from the control and T2D serum pools (50 mg) were labeled with Cy3 and Cy5, respectively, and mixed for 2-DE analysis as previously described. Greenish (Cy3) and reddish (Cy5) spots indicate differentially expressed proteins in the control and T2D serum pools respectively.

As shown in Fig 2A, sera from the rats were categorized as the control group or the T2D group. Serum samples from individual rats were initially analyzed by 1-DE on 12.5% SDS-PAGE. Unsurprisingly, there was no significant difference in the protein patterns among the samples or between the two groups. We subsequently performed the 2-DE DIGE experiments to investigate the differentially expressed proteins between the control sample pool (Cy3 labeled) and the T2D sample pool (Cy5 labeled). Although no significant distinction was noted, DIGE technology enables the detection of minor changes in protein expression between two samples. Compared to the control sample pool, seven protein targets were found differentially expressed in the T2D sample pool (Fig 2B). As identified by MS (Table 2), some albumin cognates (gi|158138568, spots 1–5) and transthyretin (TTR; gi|136467, spot 7) were found exclusively in the control sample pool, whereas vitamin D-binding protein (DBP) precursor was downregulated in the T2D sample pool (gi|203941, spot 6).

**Table 2:** MS/MS identification of the differentially expressed proteins, phosphoproteins, glycoproteins between the control and T2D serum pools.

Spot No.	Accession No.	Protein Description	Theoretical *M*_r_ / p*I*	MOWSE score	Expression (T2D/Control)^a^
**Differentially expressed proteins**					
**1**	**gi|158138568**	**albumin**	**70.7/6.09**	**217**	**Control only**
**2**	**gi|158138568**	**albumin**	**70.7/6.09**	**217**	**Control only**
**3**	**gi|158138568**	**albumin**	**70.7/6.09**	**286**	**Control only**
**4**	**gi|158138568**	**albumin**	**70.7/6.09**	**252**	**Control only**
**5**	**gi|158138568**	**albumin**	**70.7/6.09**	**319**	**Control only**
**6**	**gi|203941**	**vitamin D-binding protein precursor**	**55.1/6.76**	**370**	**-9.8**
**7**	**gi|136467**	**RecName: Full = Transthyretin; AltName: Full = Prealbumin; AltName: Full = TBPA; Flags: Precursor**	**15.8/5.77**	**88**	**Control only**
**Differentially expressed phosphoproteins**					
**1**	**gi|12831225**	**murinoglobulin 1**	**165.2/5.68**	**236**	**-1.74**
	gi|83816939	alpha-1-inhibitor III	163.67/5.7	208	-1.74
**2**	**gi|15485713**	**complement inhibitory factor H**	**144.8/6.52**	**102**	**4.88**
**3**	**gi|112889**	**Alpha-1-antiproteinase**	**46.3/5.7**	**278**	**2.24**
**4**	**gi|61556986**	**transferrin**	**76.3/7.14**	**111**	**-3.65**
	gi|149031970	rCG50690	21.2/5.00	98	-3.65
**5**	**gi|1854476**	**transferrin**	**78.5/6.94**	**102**	**-1.75**
**6**	**gi|114145409**	**type II keratin Kb15**	**57.9/8.04**	**134**	**-1.86**
	gi|109482941	PREDICTED: similar to keratin complex 2, basic, gene 6a isoform 1	59.7/8.91	115	-1.86
**7**	**gi|231468**	**RecName: Full = Alpha-2-HS-glycoprotein; AltName: Full = Fetuin-A; AltName: Full = Glycoprotein PP63; AltName: Full = 59 kDa bone sialic acid-containing protein; Short = BSP; Flags: Precursor**	**38.7/6.05**	**61**	**1.43**
**8**	**gi|205055**	**keratin K5**	**9.39/5.34**	**55**	**-1.59**
**9**	**gi|114145409**	**type II keratin Kb15**	**57.9/8.04**	**108**	**-5.19**
	gi|81891716	RecName: Full = Keratin, type II cytoskeletal 1; AltName: Full = Cytokeratin-1; Short = CK-1; AltName: Full = Keratin-1; Short = K1; AltName: Full = Type II keratin Kb1	65.2/8.00	70	-5.19
**10**	**gi|124028612**	**RecName: Full = Serum albumin; Flags: Precursor**	**70.7/6.09**	**535**	**1.91**
**11**	**gi|109482941**	**PREDICTED: similar to keratin complex 2, basic, gene 6a isoform 1**	**59.4/8.91**	**112**	**2.26**
**12**	**gi|205055**	**keratin K5**	**9.39/5.34**	**67**	**-4.40**
**13**	**gi|57012358**	**keratin 73**	**61.0/8.17**	**191**	**-2.83**
	gi|114145409	type II keratin Kb15	57.9/8.04	138	-2.83
**14**	**gi|124028612**	**RecName: Full = Serum albumin; Flags: Precursor**	**70.7/6.09**	**201**	**-2.31**
	gi|6981420	protease, serine, 2	26.6.4.17	51	-2.31
**Differentially expressed glycoproteins (ConA resin purified)**					
**1**	**gi|158138568**	**albumin [Rattus norvegicus]**	**68.7/6.09**	**609**	**-1.81**
	gi|758263	major acute phase alpha-1 [Rattus norvegicus]	47.0/6.00	585	-1.81
	gi|57526868	T-kininogen II precursor [Rattus norvegicus]	47.7/5.94	384	-1.81
	gi|116597	RecName: Full = Complement C3; Contains: RecName: Full = Complement C3 beta chain; Contains: RecName: Full = Complement C3 alpha chain; Contains: RecName: Full = C3a anaphylatoxin; Contains: RecName: Full = Complement C3b alpha' chain; Contains: RecName: Full	186.3/6.12	119	-1.81
	gi|57231	unnamed protein product [Rattus norvegicus]	45.2/5.48	117	-1.81
	gi|91942	alpha-1 proteinase inhibitor III, hepatic clone AF7—rat (fragment)	86.7/5.81	55	-1.81
	gi|2499467	RecName: Full = Complement component C9; Flags: Precursor	62.2/5.51	42	-1.81
**2**	**gi|61556986**	**transferrin [Rattus norvegicus]**	**76.3/7.14**	**1140**	**T2D only**
**3**	**gi|149018747**	**rCG25416, isoform CRA_b [Rattus norvegicus]**	**65.1/6.41**	**1280**	**-1.88**
	gi|16758014	hemopexin [Rattus norvegicus]	51.3/7.58	115	-1.88
**4**	**gi|17865327**	**fetuin B [Rattus norvegicus]**	**41.5/6.71**	**173**	**Control only**
**5**	**gi|455105**	**fibrinogen B beta chain**	**54.3/7.89**	**484**	**-8.48**
	gi|1351954	RecName: Full = Beta-2-glycoprotein 1; AltName: Full = Beta-2-glycoprotein I; Short = Beta(2)GPI; Short = B2GPI; AltName: Full = Apolipoprotein H; Short = Apo-H; Flags: Precursor	33.2/8.59	140	-8.48
	gi|1326412	IgM heavy chain variable region {VH-D-JH region} [rats, LEW, HAR-1 hybridoma cells, Peptide Partial, 121 aa]	13.4/8.96	88	-8.48
**6**	**gi|158186678**	**fibrinogen beta chain [Rattus norvegicus]**	**54.2/7.90**	**865**	**Control only**
	gi|229196	fibrinopeptide B	1.5/4.43	103	Control only
	gi|1326412	IgM heavy chain variable region {VH-D-JH region} [rats, LEW, HAR-1 hybridoma cells, Peptide Partial, 121 aa]	13.4/8.96	76	Control only
**8**	**gi|455105**	**fibrinogen B beta chain**	**54.3/7.89**	**522**	**5.21**
	gi|121041	RecName: Full = Ig gamma-1 chain C region	35.9/6.43	174	5.21
	gi|16758014	hemopexin [Rattus norvegicus]	51.3/7.58	139	5.21
	gi|1326412	IgM heavy chain variable region {VH-D-JH region} [rats, LEW, HAR-1 hybridoma cells, Peptide Partial, 121 aa]	13.4/8.96	139	5.21
**9**	**gi|33086640**	**Ba1-647 [Rattus norvegicus]**	**42.4/6.11**	**507**	**-3.51**
	gi|149038142	haptoglobin, isoform CRA_a [Rattus norvegicus]	16.1/5.37	336	-3.51
	gi|83816939	alpha-1-inhibitor III [Rattus norvegicus]	163.7/5.7	213	-3.51
**10**	**gi|33086640**	**Ba1-647 [Rattus norvegicus]**	**42.4/6.11**	**637**	**-1.47**
	gi|71051724	LOC297568 protein [Rattus norvegicus]	78.8/5.45	292	-1.47
	gi|91942	alpha-1 proteinase inhibitor III, hepatic clone AF7—rat (fragment)	86.7/5.81	113	-1.47
	gi|21955142	pregnancy-zone protein [Rattus norvegicus]	167.1/6.46	80	-1.47
	gi|112889	RecName: Full = Alpha-1-antiproteinase; AltName: Full = Alpha-1-antitrypsin; AltName: Full = Alpha-1-proteinase inhibitor; Flags: Precursor	46.7/5.10	66	-1.47
**11**	**gi|33086640**	**Ba1-647 [Rattus norvegicus]**	**42.4/6.11**	**523**	**1.98**
	gi|83816939	alpha-1-inhibitor III [Rattus norvegicus]	163.7/5.7	190	1.98
**12**	**gi|8393197**	**C-reactive protein, pentraxin-related [Rattus norvegicus]**	**25.5/4.89**	**102**	**-2.31**
**13**	**gi|157830289**	**Chain A, Crystal Structure Of The Ankyrin Binding Domain Of Alpha-Na, K-Atpase As A Fusion Protein With Glutathione S-Transferase**	**29.5/6.33**	**79**	**-1.61**
**14**	**gi|157830289**	**Chain A, Crystal Structure Of The Ankyrin Binding Domain Of Alpha-Na, K-Atpase As A Fusion Protein With Glutathione S-Transferase**	**29.5/6.33**	**58**	**-1.18**
**Differentially expressed glycoproteins (Lentil resin purified)**					
**1**	**gi|203280**	**carboxylesterase precursor (EC 3.1.1.1)**	**59.0/5.51**	**750**	**3.75**
	gi|71051724	LOC297568 protein [Rattus norvegicus]	78.8/5.45	262	3.75
	gi|16758014	hemopexin [Rattus norvegicus]	51.3/7.58	238	3.75
	gi|91942	alpha-1 proteinase inhibitor III, hepatic clone AF7—rat (fragment)	86.1/5.81	166	3.75
	gi|205085	LMW T-kininogen I precursor	47.4/6.29	153	3.75
	gi|12831225	murinoglobulin 1 [Rattus norvegicus]	165.2/5.68	130	3.75
**2**	**gi|220698**	**contrapsin-like protease inhibitor (CPi-21) [Rattus norvegicus]**	**46.4/5.3**	**879**	**2.21**
	gi|57294	unnamed protein product [Rattus norvegicus]	46.5/5.31	851	2.21
	gi|2507387	RecName: Full = Serine protease inhibitor A3L; Short = Serpin A3L; AltName: Full = Contrapsin-like protease inhibitor 3; AltName: Full = CPI-23; AltName: Full = Serine protease inhibitor 1; Short = SPI-1; Flags: Precursor	46.2/5.48	800	2.21
	gi|231468	RecName: Full = Alpha-2-HS-glycoprotein; AltName: Full = Fetuin-A; AltName: Full = Glycoprotein PP63; AltName: Full = 59 kDa bone sialic acid-containing protein; Short = BSP; Flags: Precursor	38.0/6.05	178	2.21
**3**	**gi|58865630**	**serine (or cysteine) peptidase inhibitor, clade C (antithrombin), member 1 [Rattus norvegicus]**	**52.2/6.18**	**415**	**3.13**
	gi|231468	RecName: Full = Alpha-2-HS-glycoprotein; AltName: Full = Fetuin-A; AltName: Full = Glycoprotein PP63; AltName: Full = 59 kDa bone sialic acid-containing protein; Short = BSP; Flags: Precursor	38.0/6.05	377	3.13
	gi|12831225	murinoglobulin 1 [Rattus norvegicus]	165.2/5.68	225	3.13
	gi|149049551	rCG29619, isoform CRA_a [Rattus norvegicus]	163.6.5.68	198	3.13
	gi|57233	unnamed protein product [Rattus norvegicus]	45.7/5.37	153	3.13
	gi|57231	unnamed protein product [Rattus norvegicus]	45.2/5.48	149	3.13
	gi|91942	alpha-1 proteinase inhibitor III, hepatic clone AF7—rat (fragment)	86.7/5.81	109	3.13
	gi|58865362	serine (or cysteine) peptidase inhibitor, clade F, member 2 [Rattus norvegicus]	54.9/5.74	82	1.98
**4**	**gi|112889**	**RecName: Full = Alpha-1-antiproteinase; AltName: Full = Alpha-1-antitrypsin; AltName: Full = Alpha-1-proteinase inhibitor; Flags: Precursor**	**46.1/5.7**	**724**	**1.98**
	gi|71051724	LOC297568 protein [Rattus norvegicus]	78.8/5.45	155	1.98
	gi|12831225	murinoglobulin 1 [Rattus norvegicus]	165.2/5.68	152	1.98
	gi|68052028	RecName: Full = Alpha-1-antitrypsin; AltName: Full = Alpha-1 protease inhibitor; AltName: Full = Alpha-1-antiproteinase; Flags: Precursor	45.8/5.55	105	1.98
	gi|91942	alpha-1 proteinase inhibitor III, hepatic clone AF7—rat (fragment)	86.7/5.81	105	1.98
	gi|57233	unnamed protein product [Rattus norvegicus]	45.7/5.37	64	1.98
	gi|68052097	RecName: Full = Alpha-1-antiproteinase; AltName: Full = Alpha-1-antitrypsin; AltName: Full = Alpha-1-proteinase inhibitor; Flags: Precursor	46.2/5.44	62	1.98
**6**	**gi|149018747**	**rCG25416, isoform CRA_b [Rattus norvegicus]**	**65.1/6.41**	**923**	**-1.73**
	gi|61556986	transferrin [Rattus norvegicus]	76.3/7.14	921	-1.73
**7**	**gi|112889**	**RecName: Full = Alpha-1-antiproteinase; AltName: Full = Alpha-1-antitrypsin; AltName: Full = Alpha-1-proteinase inhibitor; Flags: Precursor**	**46.1/5.7**	**447**	**4.31**
	gi|71043608	Cd5 molecule-like [Rattus norvegicus]	37.8/5.26	355	4.31
	gi|50657404	murinoglobulin 2 [Rattus norvegicus]	161.5/6.15	337	4.31
	gi|83816939	alpha-1-inhibitor III [Rattus norvegicus]	163.7/5.7	305	4.31
	gi|12831225	murinoglobulin 1 [Rattus norvegicus]	165.2/5.68	238	4.31
	gi|122065184	RecName: Full = Fibrinogen gamma chain; Flags: Precursor	50.6/5.62	169	4.31
	gi|21955142	pregnancy-zone protein [Rattus norvegicus]	167.1/6.46	145	4.31
**8**	**gi|112889**	**RecName: Full = Alpha-1-antiproteinase; AltName: Full = Alpha-1-antitrypsin; AltName: Full = Alpha-1-proteinase inhibitor; Flags: Precursor**	**46.1/5.7**	**263**	**2.71**
	gi|122065184	RecName: Full = Fibrinogen gamma chain; Flags: Precursor	50.6/5.62	243	2.71
	gi|71043608	Cd5 molecule-like [Rattus norvegicus]	37.8/5.26	186	2.71
	gi|83816939	alpha-1-inhibitor III [Rattus norvegicus]	163.7/5.7	87	2.71
	gi|68052028	RecName: Full = Alpha-1-antitrypsin; AltName: Full = Alpha-1 protease inhibitor; AltName: Full = Alpha-1-antiproteinase; Flags: Precursor	45.8/5.55	84	2.71
	gi|50657404	murinoglobulin 2 [Rattus norvegicus]	161.5/6.15	69	2.71
**9**	**gi|33086640**	**Ba1-647 [Rattus norvegicus]**	**42.4/6.11**	**363**	**2.23**
	gi|21955142	pregnancy-zone protein [Rattus norvegicus]	167.1/6.46	108	2.23

For protein spots with multiple identifications, the information of proteins with a highest MOWSE score is shown in bold text.

### Proteomic analysis of post-translational modifications

We further analyzed differences in the phosphoproteomes and glycoproteomes between the control and T2D sample pools. It should be noted that phosphoproteins and glycoproteins account for a very insignificant portion of the total serum proteins. After performing the IMAC phosphoprotein purification and the ConA and lentil glycoprotein enrichment procedures, the phosphoproteins and glycoproteins recovered accounted for fewer than 0.5 and 2% of the total serum proteins, respectively.

**Fig 3:**
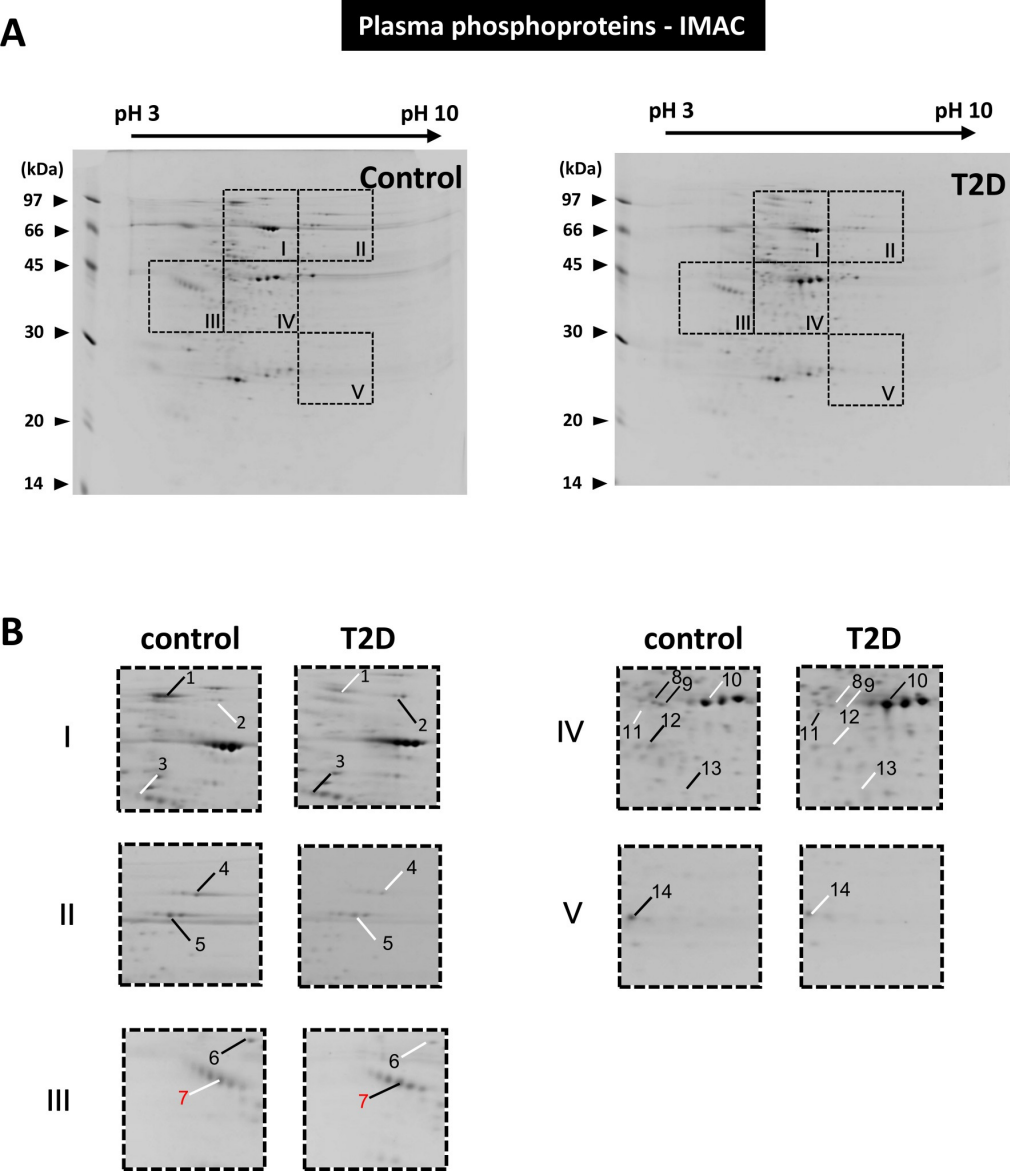
Differentially expressed phosphoproteins in the control and T2D serum pools. (A) The results of the 2-DE analysis of purified phosphoproteins from the control and T2D serum pools. The dashed lines highlight five image sections of interest. (B) Two-fold enlargement of the image sections indicated in **Fig 3A**. In comparison to the paired images, protein spots that exhibited relatively high or low expression are indicated by black and white lines, respectively.

The 2-DE patterns of the purified serum phosphoproteins were quite different from the total serum protein patterns (compare Figs 2B and 3A). Between the control and T2D groups, 14 differentially expressed phosphoproteins were identified (Fig 3B). As identified by MS (Table 2), proteins such as complement inhibitory factor H (gi|15485713, spot 2) and alpha-1-inhibitor III (gi|83816939, spot 3) were found upregulated in the T2D sample pool whereas proteins such as murinoglobulin 1 (gi|12831225, spot 1) and transferrin (gi|61556986, spot 4 and gi|1854476, spot 5) were down regulated in the T2D sample pool. Due to the limited amount of samples, MS identification of phosphopeptides was lacking for some proteins (data not shown). Nevertheless, the phosphorylation of most of the serum proteins identified has been reported.

**Fig 4:**
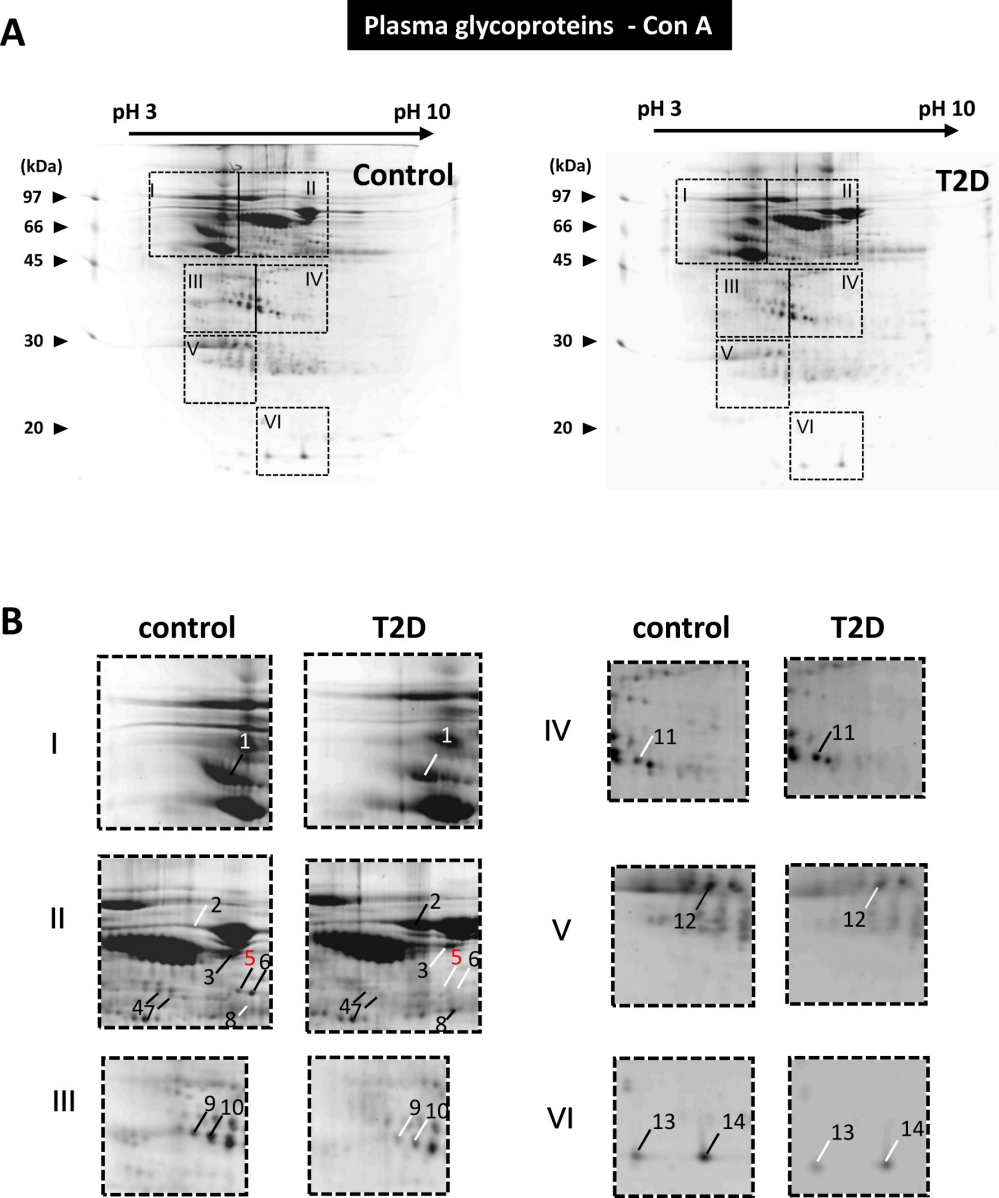
Differentially expressed glycoproteins in the control and T2D serum pools. (A) The results of the 2-DE analysis of ConA resin-enriched glycoproteins from the control and T2D serum pools. The dashed lines highlight six image sections of interest. (B) Two fold enlargement of the image sections indicated in **Fig 4A**. In comparison to the paired images, protein spots that exhibited relatively high or low expression are indicated by black and white lines, respectively.

**Fig 5:**
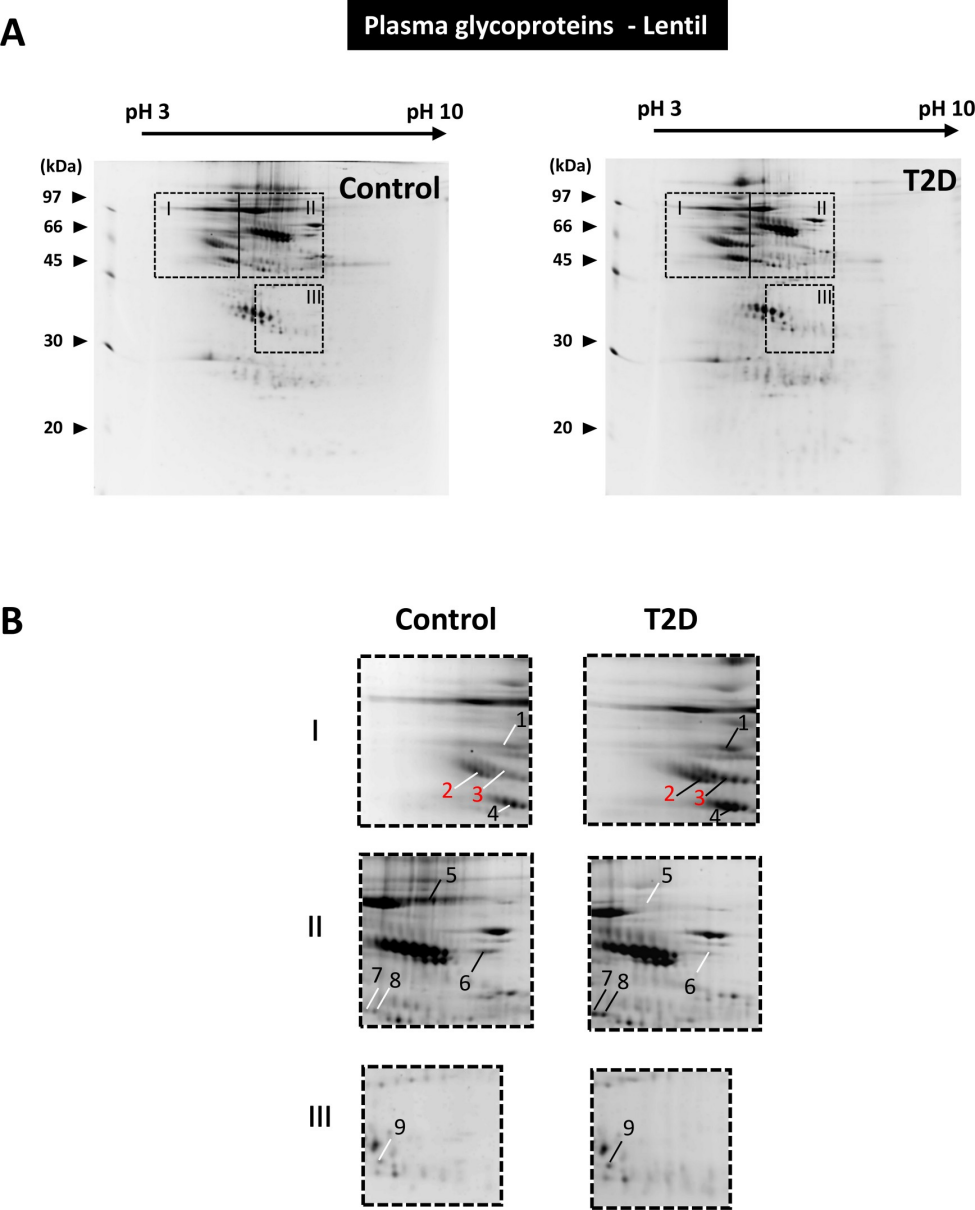
Differentially expressed glycoproteins in the control and T2D serum pools. (A) The results of the 2-DE analysis of Lentil resin-enriched glycoproteins from the control and T2D serum pools. The dashed lines highlight three image sections of interest. (B) Two-fold enlargement of the image sections indicated in **Fig 5A**. In comparison to the paired images, protein spots exhibited relatively high or low expression are indicated by black and white lines, respectively.

Both ConA from *Canavalia ensiformis* and lectin from *Lens culinaris* (Lentil) have an affinity to terminal D-mannosyl, D-glucosyl and/or L- fucosyl residues. Here, the ConA and lentil resins were used to enrich glycoproteins from rat serum samples. The two affinity chromatography methods demonstrated distinct glycoprotein enrichment results (compare Figs 4A and 5A). When the equivalent amount of serum sample was applied, the ConA affinity chromatography recovered a greater number and more diverse serum proteins. The identities of 23 differentially expressed proteins from the two enriched glycoprotein samples were determined by MS (Table 2). For example, using the ConA enrichment method, transferrin (gi|61556986, spot 2) was found exclusively in the T2D group, whereas fibrinogen B beta chain (gi|455105, spots 5,6 and 8) and Ba1-647 (gi|33086640, spots 9 and 10) were found downregulated in the T2D groups.

### Systems biology analysis

From 131 GI retrieved from our proteomics data, 37 unique proteins were selected for the preliminary IPA analysis. Of 37 proteins, 24 proteins with stringent consistency in the direction of protein expression were subjected to the refined IPA analysis. As a result, the differential expression of the identified protein markers implied most relevant biological functions in the two networks (Table 3, upper part). The top scoring network (score = 31) suggested antigen presentation, humoral immune response, and inflammatory response, whereas the second highest scoring network suggested lipid metabolism, molecular transport, and small molecular biochemistry. On the other hand, renal nephritis (P = 2.66E-05-2.74E-03), cardiac infarction (P = 1.37E-03-8.19E-03) and liver hyperplasia/hyperproliferation (P = 1.37E-03-1.37E-03) were organs the most likely to be involved in toxicology (Table 3, lower part).

**Table 3:** Possible protein networks and involved biological functions suggested by the analysis of systems biology software.

**(A) Most possible protein networks**						
**Suggested** **Network**	**Score**	**Focus** **Molecules**	**Top Suggested Functions**			
1	31	13	Antigen Presentation	Humoral Immune Response		Inflammatory Response
2	17	8	Lipid Metabolism		Molecular Transport	Small Molecule Biochemistry
**(B) Most possible tissue/organ toxicology**						
**Categories**			**P-value**		**Identified molecules**	
Renal Nephritis			2.66E-05-2.74E-03		CRP, CFH	
Renal Dysfunction			1.85E-04-1.85E-04		SERPINC1, C3	
Cardiac Infarction			1.37E-03-8.19E-03		SERPINC1, CRP	
Liver Hyperplasia/Hyperproliferation			1.37E-03-1.37E-03		C3	
Renal Damage			1.37E-03-3.1E-02		C3, CFH, GC	

(A) The top 2 related protein networks and correspondingly involved biological functions suggested by the Ingenuity Pathway Analysis (IPA) software. (B) The top 5 related tissue/organ toxicology suggested by IPA.

**Fig 6:**
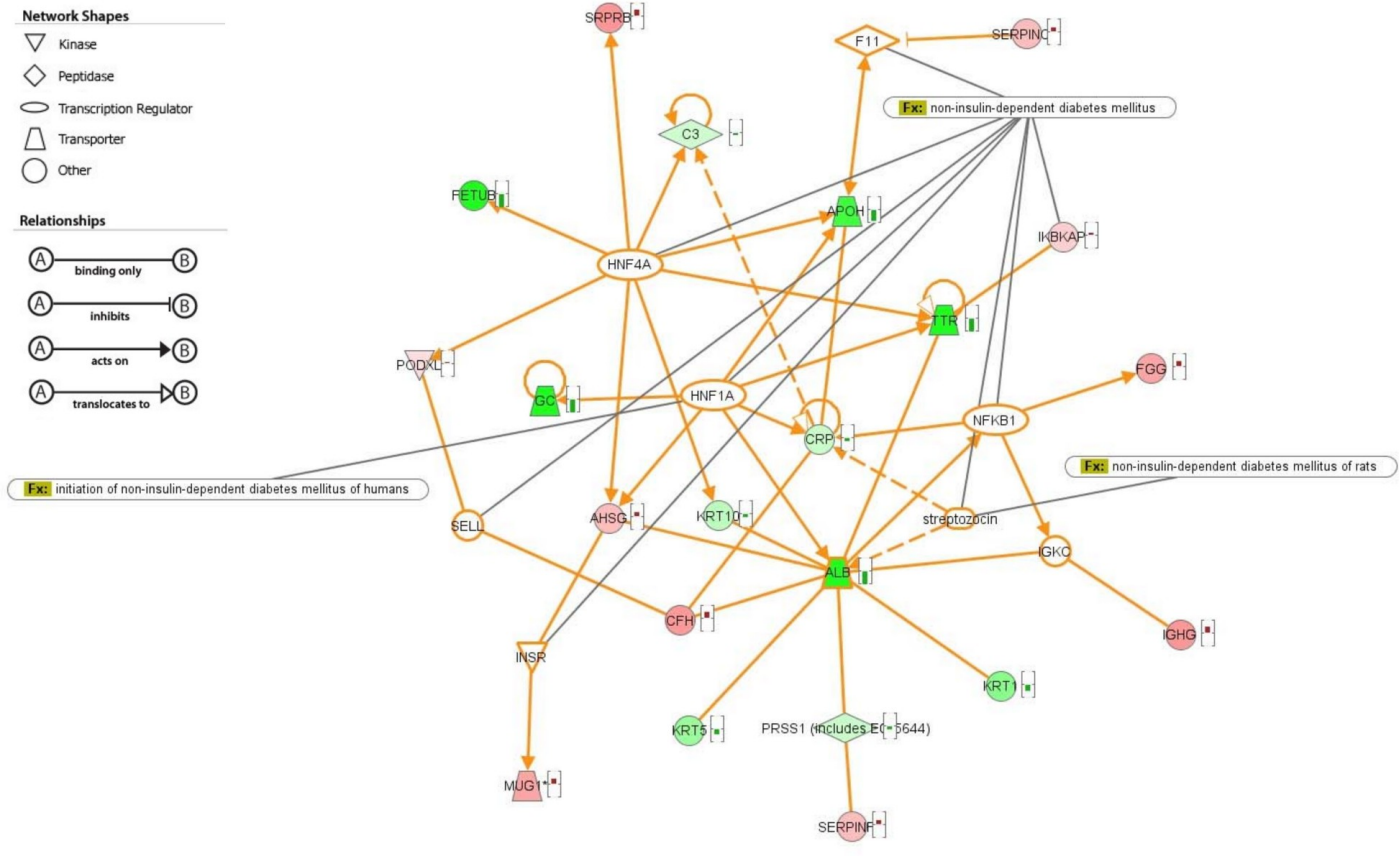
Association between differentially expressed proteins and pathways related to non-insulin dependent diabetes. Associated pathways of 24 proteins were linked with additional 7 proteins selected from IPA database with the association of non-insulin dependent diabetes. Green color labels indicate the increment of serum protein in diabetic condition. Red color labels indicates the reduction of serum protein in diabetic condition. Locations of proteins and associations of proteins were shown by indicated drawings.

To establish the association of the 24 proteins with “non-insulin-dependent diabetes mellitus”, 7 out of 1610 molecules in IPA database were added into the network in order to complete the pathways. Those proteins are coagulation factor XI (F11), hepatocyte nuclear factor 1 homeobox A (HNF1A), hepatocyte nuclear factor 4alpha (HNF4A), immunoglobulin kappa constant (IGKC), insulin receptor (INSR), nuclear factor of kappa light polypeptide gene enhancer in B-cells 1 (NFKB1), and selectin L (SELL). The result were shown in Fig 6. Albumin (ALB) appeared to be the transporter with the most interaction counterparts. HNF4A is the transcriptional factor which has the most proteins related to it.

## Discussion

Current experimental diabetes model presented the typical features of T2D, including hyperglycemia, glucose intolerance, peripheral insulin resistance, and the failure of beta-cell compensation. The apparent reduction in body weight in the T2D rats was likely owing to the recent onset of diabetes. The increase in serum triglyceride, total cholesterol, and HbAlc% in the T2D rats indicates both hyperlipidemia and the underlying development of diabetic complications. According to our pathohistological analysis, hepatic steatosis clearly occurs in the T2D rats. In addition, liver fibrogenesis appears to develop along with the increase in serum GPT levels, which indicated liver damage. Moreover, our T2D rats exhibited frequent urination and thirst (data not shown). Renal histology analysis also demonstrated the renal hypertrophy based on an increase in glomerular volume. The observed pathological conditions agreed with previous findings involving STZ-induced diabetic rats that were due to chronic oxidative and nitrosative stress [20, 21].

In general, serum proteomics without depletion of abundant proteins could only identify 3 serum proteins differentially expressed in type 2 diabetics. In contrast, after the application of protein enrichment technique, the number of identified protein targets could increase 3–5 folds. Therefore, the resolution of serum proteomics can be effectively improved by the current strategy. Interestingly, multiple albumin protein spots in 2D-DIGE clearly indicate the undergoing differential PTM in this protein and 19 glycation sites for albumin in diabetics have been identified and related to the differential extent of hyperglycemia in type 2 diabetics [22]. It should be noted that quantitative proteomics requires the alignment for the power of the experiment to reduce the potential variance in serum pooling strategies [23]. Alternatively, differential expressed protein of interest could be further quantified in individual serum samples by ELISA or western blots if required anti-bodies are available.

Based on our first set of serum proteomic investigations using 2D-DIGE techniques, the expression of albumin, TTR, and DBP were all downregulated in the T2D rats. In terms of TTR, instead of serving as a carrier of the thyroid hormone thyroxine (T4) and retinol, this protein has been proposed to play a positive role in insulin secretion via the disruption of intracellular calcium concentrations in beta cells [24]. Interestingly, elevated TTR levels in insulin-resistance ob/ob mice was also reported [25]. In our current investigation, the reduction in serum TTR expression in the T2D rats actually correlates with the results of decreased HOMA-B% and the loss of the beta-cell compensation mechanism.

The importance of vitamin D metabolism in the pathogenesis of T2D has been suggested due to the linkage of vitamin D deficiency and T2D [26]. DBP, known as a group-specific component-globulin, essentially binds to vitamin D and its metabolites to transport them to the target tissues [27]. Significant enhancement in the excretion of DBP has been suggested as the mechanism behind vitamin D deficiency in type 1 diabetes [28]. In the present study, a reduction in DBP levels in the T2D rats was also evident, supporting the role of DBP in vitamin D deficiency in diabetes mellitus. However, considering DBP is essentially synthesized in the liver [27], whether the reduction of serum DBP in the T2D rats results from enhanced excretion from the kidney or abolished protein expression due to liver damage remains to be elucidated.

As a result of the 2-D proteomics analysis involving protein samples that were enriched for different PTMs, we first found that phosphorylated keratins, podocalyxin-like protein, and transferrin were downregulated in the T2D rats. The keratins identified in this study were either basic type I (K1-K8) or acidic type II keratins (K9-K20, K73). Although keratin functions are poorly understood, limited evidence indicates that keratin phosphorylation has a protective role against hepatotoxin-induced liver injury [29]. Consistently, the expression of phosphorylated keratins was reduced in the T2D rats with liver injury.

Podocalyxin-like 1 is a transmembrane sialomucin and is highly expressed on the apical surface of glomerular epithelial cells and podocytes [30]. Studies involving knockouts have demonstrated that podocalyxin is required to maintain kidney development and podocyte function [31]. Transferrin is a circulating iron-binding glycoprotein that is mainly synthesized by the liver [32]. In diabetes, decreased plasma transferrin concentrations could lead to an increase in lipid peroxidation by enhancing the pro-oxidant effects of iron [33].

In contrast, the phosphorylation of alpha-2-HS-glycoprotein (AHSG), also known as fetuin-A, was upregulated in the T2D rats. AHSG belongs to the family of negative acute phase proteins and is secreted from the liver. The mature form of circulating AHSG has multiple PTMs including phosphorylation [34]. As a human homolog of the phosphorylated rat hepatic glycoprotein (pp63), AHSG also acts as a physiological inhibitor of insulin receptor-tyrosine kinase [35]. As well, the serum levels of AHSG are positively correlated with steatosis and insulin resistance [36, 37]. Consistently, the expression levels of phosphorylated AHSG elevated in the T2D rats with increased HOMA-IR and hepatic steatosis.

In terms of glycosylated proteins in the control and T2D rats, differential expression of serum proteins isolated from ConA or LCH columns suggested that the glycosylation process was altered in the T2D rats. Such an observation appears to be supported by the finding that glycoproteins detected in diabetic serum exhibit increased fucose content [13]. In addition, a previous investigation also demonstrated that α1,6-fucosylation in the db/db mice is changed significantly due to an elevation of hepatic α1,6-fucosyltransferase mRNA expression [15]. Therefore, future studies of the pathological roles of glycosylation and related associated enzymes might advance our knowledge of the pathogenesis of T2D and its complications.

Interestingly, the majority of proteins with PTMs that were altered in sera from the T2D rats were secreted by the liver. The liver is known to play an important physiological role in the regulation of glucose and lipid metabolism. In contrast, hepatic steatosis is a common pathological characteristic that occurs during obesity and T2D and is associated with insulin resistance, dyslipidemia, and inflammation [38]. According to a recent study published by Meex et. al., the hepatic protein secretome is altered in primary hepatocytes isolated from mice with hepatic steatosis that were fed a high-fat diet. Comparing their results with our proteomic data, similar pathways that were analyzed based on identified targets in both studies were pathways involved in regulating systemic glycemic control (e.g., ASHG, fetuin-B, and alpha1-antitrypsin), lipid or heme metabolism (e.g., apolipoprotein H and hemopexin), and immune response (e.g., complement factor, macroglobulin, IκB, and alpha-1-inhibitor III); [39, 40].

Among those targets, fetuin-B belongs to the superfamily of cysteine protease inhibitors with little homology to AHSG [41]. Although both hepatic-secreted proteins are positively associated with hepatic steatosis, insulin resistance, and diabetes, the fetuin-B mechanism of action is different from AHSG, which acts as an inhibitor of insulin receptors [35, 40]. In addition, apolipoprotein H (beta-2-glycoprotein I; Apo H) levels are increased in hyperlipidemic subjects and type 2 diabetics with microalbuminuria [42, 43]. Apo H is also employed as a biomarker for detecting early tubular disorder in diabetic patients in the absence of clinical proteinuria when combining two other biomarkers, which are retinol binding protein and N-acetyl-beta-D-glucosaminidase [44]. The affinity of fetuin-B and Apo H for ConA was substantially downregulated in the sera from the T2D rats. In contrast, alpha-1-antitypsin was found to protect β-cells from STZ- and cytokine-mediated apoptosis [45]. Further, the levels of alpha-1-antitypsin were downregulated in the ConA fraction but upregulated in the LCH fraction of the sera from the T2D rats.

The results of the systems biology analysis appeared to support our observations related to renal and liver toxicity in the T2D rats. Among the molecules identified, C-reactive protein (CRP) is an acute-phase protein secreted from the liver in response to inflammation, infection, and tissue damage. In a nephrotoxic nephritis (NTN) model induced by the depletion of complement C3, CRP administration in NTN mice decreases proteinuria and attenuates glomerular hypertrophy [46, 47]. Interestingly, both proteins were downregulated in the T2D rats in glycoproteome (ConA) analysis. Antithrombin III/SerpinC1 is a serine protease inhibitor with anti-coagulation effects and the heterozygous knock-out of the SerpinC1 gene in rats results in exacerbation of renal ischemia/reperfusion injury [48]. In this case, the elevation of protein levels in the glycoproteom (Lentil) was observed.

Finally, identified 24 proteins could be connected by 7 T2D-related proteins from IPA database. 3 of 7 proteins are important transcriptional factors in T2D pathogenesis [49–51]. Interestingly, both HNF1A and HNF4A directly participate in the regulation of glycosylation of serum proteins [52–54]. Therefore, further investigation is required to elucidate the underlying mechanisms in T2D.

## Conclusions

The application of PTM enriched methodology could improve the resolution of non-depleted serum proteomics analysis. Our results also indicate the important role of hepatocyte nuclear factors and hepatic secretome in the pathogenesis of type 2 diabetes and its complications. Further translational research comparing human and rodent data is the crucial process for validation to ensure the clinical relevance of identified protein targets in the future.

## Supporting information

S1 Checklist (PDF)
